# Radiation-Induced Heart Disease: Pathologic Abnormalities and Putative Mechanisms

**DOI:** 10.3389/fonc.2015.00039

**Published:** 2015-02-18

**Authors:** Neil K. Taunk, Bruce G. Haffty, John B. Kostis, Sharad Goyal

**Affiliations:** ^1^Department of Radiation Oncology, Memorial Sloan Kettering Cancer Center, New York, NY, USA; ^2^Department of Radiation Oncology, The Cancer Institute of New Jersey, Robert Wood Johnson Medical School, Rutgers University, New Brunswick, NJ, USA; ^3^Department of Medicine, The Cardiovascular Institute of New Jersey, Robert Wood Johnson Medical School, Rutgers University, New Brunswick, NJ, USA

**Keywords:** breast cancer, radiation side effects, radiation therapy, radiation fibrosis

## Abstract

Breast cancer is a common diagnosis in women. Breast radiation has become critical in managing patients who receive breast conserving surgery, or have certain high-risk features after mastectomy. Most patients have an excellent prognosis, therefore understanding the late effects of radiation to the chest is important. Radiation-induced heart disease (RIHD) comprises a spectrum of cardiac pathology including myocardial fibrosis and cardiomyopathy, coronary artery disease, valvular disease, pericardial disease, and arrhythmias. Tissue fibrosis is a common mediator in RIHD. Multiple pathways converge with both acute and chronic cellular, molecular, and genetic changes to result in fibrosis. In this article, we review the pathophysiology of cardiac disease related to radiation therapy to the chest. Our understanding of these mechanisms has improved substantially, but much work remains to further refine radiation delivery techniques and develop therapeutics to battle late effects of radiation.

## Introduction

Breast cancer is a common diagnosis in women with an estimated diagnosis of 235,000 new cases made in 2014. Annually approximately 40,000 women are expected to die from breast cancer (1). Adjuvant radiation therapy (RT) following either breast conserving surgery (BCS) or mastectomy has been shown in comprehensive meta analyses to reduce the risk of local recurrence by approximately 75%. Unfortunately, RT to the breast and chest has been associated with radiation-related morbidity and mortality that may offset some of the benefit of breast radiation. The spectrum of radiation-induced heart disease (RIHD) includes pericarditis, cardiomyopathy and myocardial fibrosis, coronary artery disease, pericardial effusions or constriction, valvular disease, and arrhythmias (2, 3). The spectrum of RIHD in patients undergoing other thoracic and mediastinal RT has been described since 1960s. Today, breast cancer patients likely constitute the largest population of patients exposed to chest radiation (4, 5). Recently published studies indicating that breast RT may pose an increased risk of heart disease have re-emphasized the importance of minimizing the heart dose (6). Modern techniques including three-dimensional planning, conformal blocking, deep-inspiration breath hold, and prone positioning, among others, have allowed the radiation oncologist to reduce the heart dose during breast RT, potentially reducing or eliminating RIHD. In this article, we review the pathophysiology of RIHD from several common pathways, and mechanisms for the specific cardiac pathologies. It is important to know that RIHD is a heterogenous group of pathologic abnormalities. Substantial work has been performed in histologic description, but further characterization of the biochemical pathways is required.

## Normal Heart Tissue Anatomy

The heart comprised three layers of tissue: endocardium, myocardium, and epicardium. The epicardium is superficial outer layer of the heart composed of a sheet of mesothelial cells. It is also considered the visceral layer of the serous pericardium. The epicardium is responsible for producing pericardial fluid that provides lubrication between the inner serous and outer fibrous pericardium and protection of the heart from external contusion. Pericardial disease includes pericarditis (inflammation of the pericardium), pericardial effusion (fluid accumulation in the pericardial sac), cardiac tamponade (pericardial effusion leading to hemodynamic compromise), constrictive pericarditis, and other less common pathologies (4, 7–11). The endocardium most closely resembles endothelial tissue and lines the inner surface of the heart. Endothelial cells modulate the function of the cardiac myocytes in the underlying myocardium. Ventricular endocardium also contains fibers of the cardiac conduction system. The myocardium is a highly vascular tissue with a capillary density approaching 2800 capillaries per mm^2^; capillary density of skeletal muscle is approximately 350 capillaries per mm^2^. Capillaries surround individual myocytes completely and normally are always open to perfusion. Prior to initiation of an action potential, cardiac myocytes are in a resting, well-perfused state. The action potential causes a series of processes resulting in actin-myosin crossbridging and contraction. Normally, spontaneous phase 4 depolarization in cells of the sinoatrial node, the most rapid site of rhythmic discharge, initiates atrial depolarization that propagates via the atrioventricular nodes, to the His–Purkinje fibers, and to the ventricular myocytes. The myocardial blood supply is critical and relies on a developed arteriolar capillary system as there are no major vessels that course through this tissue. Any radiation-induced damage to the vascular endothelial cells that line the myocardial capillaries can result in decreased myocardial perfusion and poor contractility (12–14).

The major blood supply to the heart is from the coronary arteries. The right and left coronary arteries originate at the root of the aorta. The left coronary artery divides into the left anterior descending artery (LAD) and the left circumflex artery. The LAD is more often implicated in RT-related morbidity as it courses on the anterior surface of the heart and is most often contacted by external beam radiation (15, 16). Any disruption to arterial flow, whether by progressive occlusive disease or acute thrombotic event causing complete obstruction, can result in ischemia and potential infarct.

## Pathophysiology of Radiation-Induced Heart Damage

A major common endpoint for RIHD is tissue fibrosis. Tissue irradiation is a major model to study fibrosis (17). In a simple characterization, radiation exposure leads to endothelial cell damage and subsequent microvascular dysfunction due to fibrosis.

Radiation damage is characterized by both acute and chronic changes in cardiac tissue. Within minutes of ionizing radiation, cellular injury causes vasodilation and increased vascular permeability. Damaged endothelial cells secrete adhesion molecules and growth factors prompting activation of the acute inflammatory response. Recruited inflammatory cells secrete pro-fibrotic cytokines (17, 18). Inflammatory cytokines include monocyte chemotactic factor, tumor necrosis factor (TNF), and interleukins (IL) including IL-1, IL-6, and IL-8. The predominant cells in the acute phase are neutrophils, which become present in all layers of the heart in RT exposed regions. Within hours of RT, pro-fibrotic cytokines such as platelet-derived growth factor (PDGF), transforming growth factor β (TGF-β), basic fibroblast growth factor (bFGF), insulin-like growth factor (IGF), and connective tissue growth factor (CTGF), among others, are released (19). While some factors promote recruitment of inflammatory cells and pro-fibrotic cells, others such as IL-1, act as a tissue radioprotector (20). Matrix metalloproteinases degrade the endothelial basement membrane, allowing efficient recruitment of pro-inflammatory cells to sites of tissue injury to consume injured tissue and initiate healing. Initial microvascular damage also triggers the coagulation cascade, resulting in immediate fibrin deposition. The acute phase courses for several days after RT administration. Following this acute infiltration, there exists a quiescent period where there are no obvious microscopic changes in the tissue (21).

**Figure 1:**
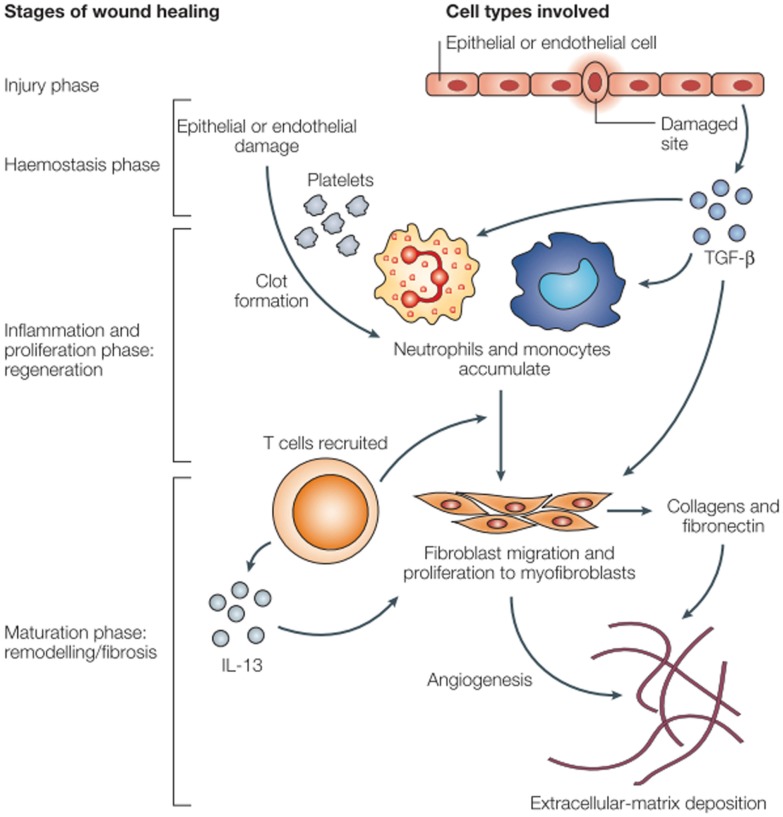
**Wound healing and fibrosis in tissue (17)**.

The acute pro-inflammatory environment is a powerful initiator of fibrosis (19). Fibroblasts are recruited from a number of different sources: derived from mesenchymal cells, recruited from bone marrow, or sourced from epithelial–mesenchymal cell transition. These changes are characterized by collagen deposition and endothelial cell proliferation. Extracellular matrix deposition by fibroblasts results in late pathologic dysfunction of myocytes, vascular endothelial cells, and the pericardium (Figure 1). Aside from the acute inflammatory response, there is an immediate expression of proto-oncogenes including c-myc and c-jun, which may prompt late fibrotic changes (22, 23). Multiple mediators ultimately result in long-term recruitment of matrix metalloproteinases, inflammatory mediators such as IL-4, IL-13, and TGF-β, and smooth muscle cell proliferation. IL-13 is a known potent fibrotic mediator produced by inflammatory T cells and in certain mouse models, IL-13 knockouts do not experience fibrosis (17). TGF-β is known as a fibroblast mediator and can induce fibroblast differentiation. TGF-β can alter the balance of extracellular matrix remodeling to induce collagen synthesis, decrease production of collagenase and other proteases, and increase the production of protease inhibitors. TGF-β has a multitude of effects and its expression is continues in irradiated tissues (24). After myofibroblasts have been activated, collagen deposition and fibroblast differentiation can continue independent of TGF-β signaling by autocrine induction (19, 22). Chronic oxidative stress with free radical production and this persistent pro-inflammatory facilitate the development of late atherosclerotic disease.

It is already well-known that tissue irradiation ultimately leads to fibrosis; however, radiation changes the biology of pro-fibrotic cells. Ionizing radiation induces premature differentiation of fibroblasts. In normal fibroblast differentiation, 25–35 cell division cycles are required. After ionizing radiation, progenitor fibroblasts differentiate into post-mitotic fibroblasts within 2–3 weeks, representing only 3–4 cell cycles. The lifespan of these terminally differentiated radiation-induced fibrocytes is nearly 40–45% shorter than naturally differentiated cells. These post-mitotic cells are shown to be five to eight times more active in the production of interstitial collagens I, III, and IV compared to progenitor fibroblasts. Ionizing radiation, on its own, can induce premature terminal differentiation of progenitor fibroblasts to post-mitotic fibrocytes that are more active in collagen deposition (25, 26). Myofibroblasts are permanently activated in these tissues even after repair of initial damage, unlike in normal wound repair (27). Chronic deposition of collagen and other components of other extracellular matrix components can produce a fibrotic scar reducing functionality of the affected tissue. Pathologic examination of these lesions show elevated inflammatory cells, fibroblasts, and excessive extracellular matrix, such as collagens, proteoglycans, and fibronectin.

**Figure 2:**
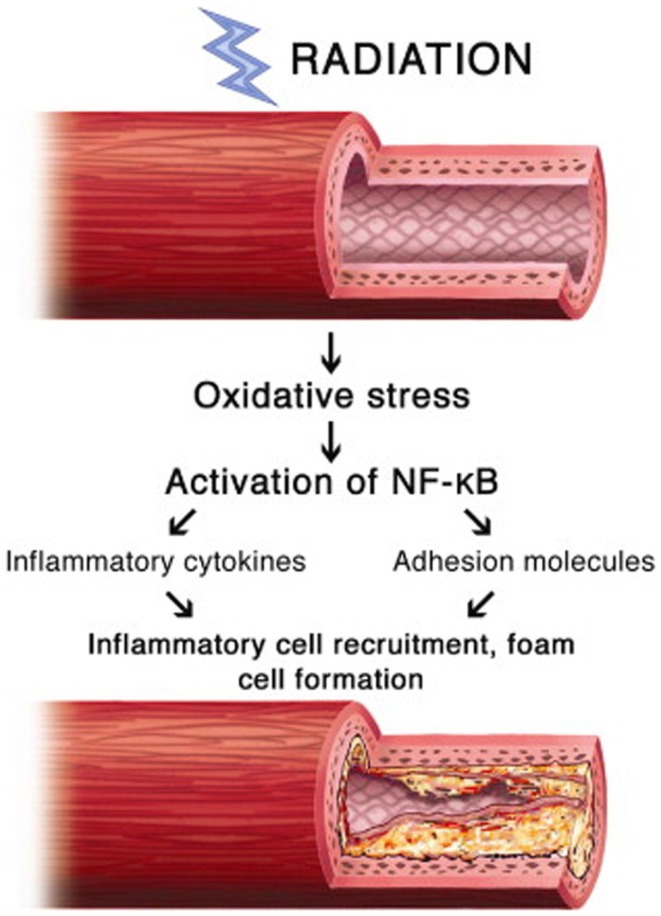
**Proposed mechanism of involvement of NF-κB in radiation-induced vascular disease (28)**.

The inflammatory pathway is likely the predominant pro-fibrotic mediator, but other pathways contribute significantly. Another mediator is chronic oxidative stress, the result of chronic free radical production. The oxidative stress simultaneously increases inflammatory mediators, proteases, and adhesion molecules, and decreases nitric oxide, a vascular protectant that blocks platelet aggregation and vascular smooth muscle proliferation. Nuclear factor-kappa B (NF-κB), a protein complex that regulates DNA transcription and is involved in cellular response to various stresses, may serve as a key link between oxidative stress and inflammatory pathways (Figure 2). In a study of irradiated human neck arteries, NF-κB is chronically upregulated locally in irradiated human arterial vascular cells anywhere from 4 to 500 weeks after treatment (28, 29).

In addition to NF-κB, other changes in gene expression mediate a pro-fibrotic environment. Chronic hypoxia from microvessel damage leads to upregulation of hypoxia inducible factor α (HIF1-α), which is another stimulator of TGF-β (19). This provides further evidence how local radiation can result in chronic gene expression changes leading to long term and late pathology (28, 29).

**Figure 3:**
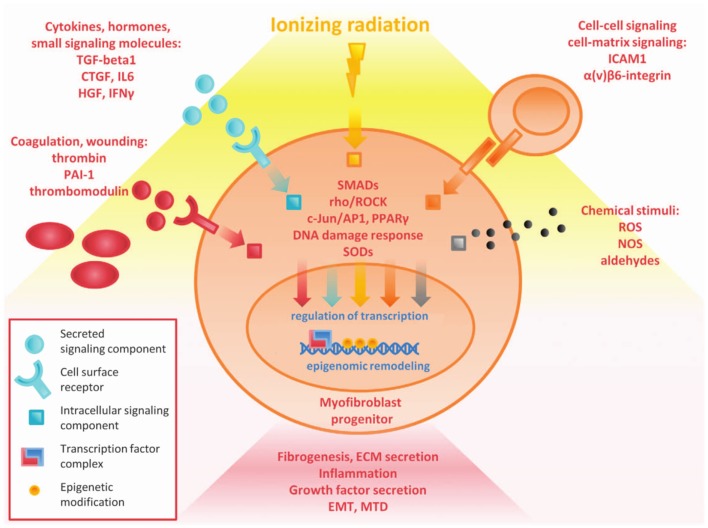
**Overview of complex pathways in tissue fibrosis (27)**.

Fibrosis is both acute and late effect of tissue irradiation. It is the result of multiple converging pathways including inflammation, oxidative stress, and chronic changes in gene expression (Figure 3). There is broad involvement of the DNA damage response, TGF-β signaling, and the chronic inflammatory pathways. Acute changes largely result from direct radiation damage and the immediate inflammatory response. Long-term changes in the tissue and characterization of characterization of epigenetic changes, altered cell signaling, and stem cell loss are critical to understanding late and persistent fibrosis (27). The incredibly complex interplay between multiple converging pathways may lead to a variety of clinical targets to combat fibrosis. However, many of these targets have pleiotropic effects leading to other toxicity, and knocking down a single pro-fibrotic pathway may not be sufficient to show clinical benefit.

## Coronary Artery Disease

**Figure 4:**
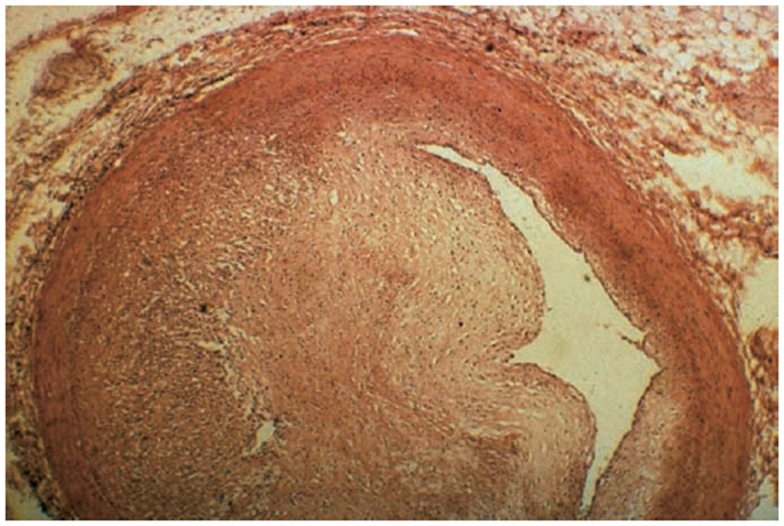
**Significant fibrosis of the left anterior descending (LAD) artery after chest radiation (58)**.

The initiation of RIHD in the coronary arteries is similar to that of most other tissues as radiation leads to microvascular damage, inflammation, and subsequent fibrosis. In general, the pathologic changes observed in RIHD are morphologically similar to atherosclerotic disease in medium and large vessels (100–500 and >500 μm, respectively) (30). In small-sized arteries, there is often subendothelial fibrosis, accumulation of acellular collagenous material in the media, and accumulation of lipid-laden macrophages (foam cells) in the intima (31). In medium-sized arteries, foam cells, fibroblasts, and collagen accumulate in the intima. Occasionally, there is acute vasculitis with a predominantly lymphocytic rich infiltrate in the media and adventitia. It is presumed this pathology is self-limited based on animal models. In one swine study with coronary and iliac arteries subjected to P^32^ endovascular brachytherapy, 51% of arterioles sampled near exposed coronary arteries and 100% of arterioles near iliac arteries had evidence of vasculitis in doses from 6 to 40 Gy at 28 days post-exposure. This was noted to be morphologically dissimilar than other systemic vasculitides (32). The smooth muscle layer in the arteries is noted to be replaced instead by fibrous tissue (33). Large arteries are not as often affected as smaller vessels, given that a large luminal diameter allows for larger plaque accumulation before clinical evidence of disease, and thick walled vessels may have more radioresistant cells. However, large radiation-associated plaques with concomitant underlying atherosclerotic disease can lead to plaque rupture and thrombosis (Figure 4).

The endothelial cells respond with inflammatory markers and adhesion molecules to recruit peripheral leukocytes with doses as little as ≥2 Gy. Once monocytes enter the subendothelial space, they may transform into activated macrophages. Activated macrophages can ingest lipids, forming fatty streak in the intima leading to early atherosclerotic lesions. Late proliferation of myofibroblasts can further the growth of these luminal-narrowing lesions. Dose of RT ≥8 Gy are associated with increased size and number of these lesions in the major arteries. In addition, the plaques that result may be more unstable and macrophage-laden (34). Unlike stable collagenous plaques, radiation-related plaques tend to grow, rupture, and lead to a myocardial infarction or cerebrovascular accident more often (35). It is important to note that these dose–response data include series from *in vitro* models and limited autopsy assessment. Although the data appear to confirm clinical suspicion, further assessment would be required particularly in the era of modern radiation techniques with different dose constraints and refined treatment planning.

The arteries affected and plaque location differs from usual atherosclerotic disease. Compared to usual atherosclerotic disease, the LAD artery tends to be often involved in RIHD. This may be due to RT biased toward involvement of the anterior chest. Lesions in RIHD tend to involve a longer length of artery than usual atherosclerotic plaques. Maximum luminal narrowing tends to be at the distal ends of the lesions, and often at arterial bifurcations (16).

Arterial fibrosis is a well-studied phenomenon in RIHD and radiation exposure is an independent risk factor for long-term cardiovascular disease. This is apparent in early stage breast cancer, Hodgkin’s disease, and other childhood cancer. There is epidemiologic evidence associating high-dose exposure with cardiac morbidity, including coronary artery disease (2, 36, 37). The dose–response relationship leading to clinically meaningful morbidity is still poorly understood, particularly with low-dose radiation exposure. Preclinical data report that dose of 2 Gy does not alter vessel phenotype in moderate-term followup and ultra-low-dose exposure of <0.5 Gy can even have anti-atherosclerotic effect (38). However, new clinical data in breast cancer survivors suggest that there is no low-dose threshold that increases the risk of RIHD (6). Conventional and three-dimensional RT (3DCRT) has given way to newer technologies such as intensity-modulated radiation therapy (IMRT) that offers increasing dose homogeneity in the target volume with potential for normal tissue sparing. It remains an open question what effect spreading of low-dose radiation with IMRT from multiple beam angles will have on atherosclerotic disease.

## Myocardial Fibrosis and Cardiomyopathy

The myocardial subunit is composed of cardiac myocytes, capillaries, and stromal tissue. Each myocardial subunit has a network of capillaries and relies on diffusion for nutrient metabolism, as there are no arterioles in the tissue. Damage to the myocardium occurs after capillary loss from radiation-induced microvasculature damage. Decrease in capillary density results in islands of hypoxia in the myocardial tissue (18). In a study by Fajardo and Stewart, it was noted that 100 days after RT exposure, there was a significant reduction in the ratio of capillaries to cardiac myocytes. There was also endothelial cell membrane alteration with subsequent microthrombus formation (34). There may be some compensatory transient capillary proliferation; however, this largely appears to be inadequate to compensate for progressive and chronic microvascular damage.

Microvascular damage also leads to inflammatory and pro-thrombotic changes. After cell damage and death, pathologic changes are indicative of progressive fibrosis replacing myocardial tissue. Chello et al. conducted an autopsy study of normal heart tissue compared to left ventricular tissue of patients with post-radiation pericarditis. In the ventricular tissue of irradiated hearts, there was a significant increase in total tissue collagen concentration compared to non-irradiated hearts, consistent with long-term fibrosis. Both Type I and Type III collagen were increased; however, there was a disproportionate increase in the amount of Type I collagen. Type I is more often found in repair tissue whereas Type III collagen is more often found acutely in granulation tissue. This may lead to decreased distension of the ventricles during filling (39).

Progressive fibrosis of the myocardium ultimately leads to decrease in tissue elasticity and distensibility, particularly after replacement with Type I collagen. This leads to reduction in ejection fraction and increase in left ventricular end-diastolic volume and reduced ejection fraction. Marks et al. conducted a study of 114 patients with left sided breast cancer designed to study changes in regional and global cardiac function using technetium-99_m_ sestamibi or tetrofosmin scans before and after breast radiation. At 6, 12, 18, and 24 months, 27, 29, 38, and 42% of patients, respectively, had new perfusion defects. These patients with perfusion defects were also more likely to have regional wall motion abnormalities (40). This suggests that microvascular damage leads to tissue fibrosis with clinically detectable cardiac function. The final outcome is that irradiation ultimately results in loss of tissue elasticity. This pertains primarily to the ventricles rather than the coronary arteries.

The cardiovascular system responds differently to RT-related myocardial damage compared with ischemia-related heart failure. In RT-unrelated myocardial damage, the body activates the sympathetic nervous system continuously, while simultaneously down-regulating β-adrenergic receptors. In contrast, RT-related myocardial damage results in no augmentation of the sympathetic nervous system in the adrenal glands, but β-receptors initially are upregulated in the heart. This upregulation of the receptors may allow the heart to stabilize cardiac output despite damage. Eventually, as damage progresses, further reductions in cardiac output occur near the onset of congestive heart failure (41–43).

Fibrosis dominates both atherosclerotic disease and myocardial damage. There is similar debate regarding the relative contribution of low-dose radiation to clinical apparent cardiac morbidity. High-dose radiation exposure to the left ventricle can be obviated by a variety of heart sparing radiation techniques such as multileaf collimator (MLC) or cerrobend blocking, deep inspiration breath hold, or prone positioning. However, some low-dose exposure is unavoidable. Cardiac perfusion imaging studies have yet to show perfusion defects in areas with low exposure (heart *D*_mean_ < 5 Gy or doses of 0–10 Gy) (44, 45). This remains a very active area of study, but it is advisable to keep heart dose as low as reasonably possible particularly in the era of cardiotoxic systemic therapy.

## Valvular Disease

Valvular disease is less well characterized compared to changes in the myocardium and coronary arteries. Fibrotic damage in the valves is unlikely related to microvascular damage as the heart valves are avascular. The damage is likely related to other myocardial disease. In one example, RT-related dilated cardiomyopathy may induce regurgitation, although the exact mechanism is poorly understood. Although valvular disease has a high incidence of pathologic changes, the majority of patients do not appear to have more than moderate clinical symptoms (33, 46). One post-mortem series of patients who received at least 35 Gy to heart indicated up to 81% (13 of 16) of patients showed evidence of valvular dysfunction and fibrosis, without or without dystrophic calcification. Specimens showed focal thickening of the valvular endocardium by elastic fibers (33). Veinot conducted a study of 27 patients with multiple cardiac tissue specimens. These patients represented breast cancer, as well as lymphomas and other thoracic cancers. A clear majority of patients experienced RT-related valvular disease with a mean dose of 46 Gy. There was a significant latent period before the development of valvular symptoms with mean time at 98 months. All the valves showed diffuse cusp or leaflet fibrosis. There were no changes indicative of chronic inflammation or neovascularization, suggesting that another RT-related mechanism drives valvular pathology. There was a spectrum of mild to severe stenosis or incompetency (46). Although available series indicate a significant percentage affected with valvular disease, the incidence may likely be lower in often used tangent breast radiation given a significantly lower dose to the heart compared to thoracic or mediastinal radiation.

## Pericardial Disease

Up to 70–90% of patients with significant mediastinal radiation exposure may have evidence of pericardial disease (33, 46). In pathologic cardiac specimens after >35 Gy to the heart in young patients aged 15–33, 15/16 had thickened pericardia. Of these, five patients had pericardial tamponade (33). Initial series of Hodgkin’s disease patients indicated up to 40% of patients experienced clinical pericarditis. Use of reduced total and daily doses, as well as conformal techniques has reduced this risk nearly to 2% (21, 47). The incidence in breast radiation is likely even lower given the limited dose to the heart compared to mediastinal radiation.

There is both acute and late pericardial injury present, driven by inflammation and immediate fibrin deposition. Initial injury to the pericardium is due to microvascular damage that leads to episodic ischemia. Tortuous and permeable neovascularization occurs in irradiated pericardium, leading to additional ischemia and late fibrosis. Additional fibrosis of venous and lymphatic channels in the heart decreases the ability to drain extracellular fluid, leading to accumulation of a fibrin-rich exudate (21). Early clinical pericardial disease is generally represented by effusions (46).

Nearly 20% of patients who experienced late significant fibrosis of the pericardium may have initially had effusions (21). Fibrinous exudates on the visceral pericardium are later replaced by fibroblasts laying down collagen, leading to long-term fibrosis of the pericardium. Normal pericardial adipose tissue is replaced by collagen and fibrin. An increase in Type I collagen deposited in the pericardium decreases diastolic compliance of the ventricles, and the pericardium can be thickened from 1 to 7 mm in severe disease after radiation (39, 46). These changes can lead to a spectrum of pericardial pathologies including acute and delayed pericarditis, pancarditis, and possible severe constrictive pericarditis, resulting in tamponade (48). In Veinot’s series, patients who were found to have significant constriction became symptomatic after 18 months, suggesting a long latent period after exposure (46).

## Cardiac Arrhythmias

Conduction system abnormalities are not as well-documented or reported as the other cardiac pathologies. Arrythmias are likely due to microvascular damage, leading to cardiac myocyte conduction abnormalities or direct damage to critical structures such as the sinoatrial or atrio-ventricular nodes. This may result in AV-nodal bradycardia or all types of heart block, including complete heart block. Right bundle branch block has been observed, due to either direct damage to the conducting myocytes or adjacent microvascular damage resulting in ischemia. In a series of three Hodgkin’s lymphoma patients treated nearly 10 years prior with mantle radiation, two of the three had partial or complete right bundle branch block before the age of 35 (49). Fibrosis of the left ventricular wall is associated with increased ventricular ectopy. Nearly 12 years after thoracic irradiation, a report of six patients all showed complete atrio-ventricular node block requiring permanent pacemaker implantation. Of the six patients, five had right bundle branch block or alternating right bundle branch block. The mean RT dose was 52 Gy (50). As expected, all of these patients had multiple other pathologies, including myocardial fibrosis, pericardial disease, and coronary artery disease. In a series of nearly 200 breast cancer survivors, a significant percentage had conduction abnormalities at both 6 months and 10 years after treatment. Nineteen percent of patients had pre-treatment conduction abnormalities, which increased to 45% at both 6 months and 10 years after therapy. The predominant changes at 6 months were T wave abnormalities in left sided breast cancer patients. At 10 months, there were fewer T wave changes, but increased ST depression. Although present in a large percentage of breast cancer patients, these changes were largely reversible and clinically insignificant (51).

Ventricular ectopic beats (VEB) are commonly seen in outpatient medicine and are often benign. These include often asymptomatic premature ventricular contractions (PVC) to more dangerous ventricular tachycardia and ventricular fibrillation. The incidence is nearly 1% in clinically normal people using electrocardiogram (ECG) detection, and up to 75% in clinically well patients using Holter monitoring (52, 53). Although chest radiation may increase the incidence of VEB, a comprehensive assessment must first be performed to rule out other exacerbating factors such as ischemic heart disease, structural heart disease, substance ingestion, or smoking.

To suggest that AV abnormalities may be related to prior RT, one series suggests the following criteria be met (1) total RT dose to the heart >40 Gy (2) latency of >10 years since RT (3) an abnormal interval ECG (4) prior pericardial involvement (5) associated cardiac or mediastinal disease (54). However, these criteria would not often be met in breast cancer survivors. In long-term survivors, vigilance will be required in patients who have experienced other RIHD pathologies or who have underlying atherosclerotic disease. There must be great care in attributing arrhythmias to RT versus competing causes.

## Future Directions

There exist clear mechanisms by which RT leads to acute and long-term changes in cardiac tissue. Pathologic changes after radiation exposure with clinical implication have been well-documented. However, there are a tremendous number of unanswered questions that will be critical in understanding, prevention, and treatment of RIHD. The bulk of damage appears to be from acute and chronic inflammatory changes, but persistent oxidative stress and genetic changes also significantly contribute. It will be important to characterize the relative contribution of each pathway to evaluate, which will be the most meaningful target of therapeutics.

High-dose radiation exposure is clearly associated with cardiac toxicity; however, the contribution of low-dose radiation is not completely characterized. In addition, it is even unclear if there is a low-dose threshold before which clinically meaningful morbidity appears. The relative contribution of high-dose and low-dose radiation exposure may be augmented further by cardiotoxic systemic therapy such as anthracycline chemotherapy, or underlying patient comorbidities such as diabetes, pre-existing heart disease, and smoking.

Given the multiple pathways leading to RIHD, there are a number of potential therapeutic targets. These include anti-inflammatory mediators, anti-fibrotics, genetic modulators, and even stem cell treatment (55–57). Studies have largely been preclinical, to date, and therapeutics are either in clinical trials or under development. However, establishment of an excellent therapeutic would likely require large numbers of patients with extensive long-term follow-up.

## Conclusion

Radiation-induced heart disease represents a collection of cardiac pathology including coronary artery disease, myocardial fibrosis, pericardial disease, arrhythmias, and valvular abnormalities. There are several common pathways involved in the development of RIHD including microvascular damage, inflammation, and fibrosis, although other pathways contribute. The interaction of multiple biochemical markers and cytokines such as TGF-β and interleukins, drive a significant portion of chronic inflammation and late fibrosis. Although there exist substantial evidence RIHD has a significant incidence and can lead to substantial morbidity, the exact mechanisms of the various RIHD pathologies are not entirely understood. The development of therapeutic targets to prevent microvascular damage, inflammation, and late fibrosis will hinge on our increased understanding of RIHD.

## Conflict of Interest Statement

The authors declare that the research was conducted in the absence of any commercial or financial relationships that could be construed as a potential conflict of interest.
